# Hyperferritinemia in the critically ill child with secondary hemophagocytic lymphohistiocytosis/sepsis/multiple organ dysfunction syndrome/macrophage activation syndrome: what is the treatment?

**DOI:** 10.1186/cc11256

**Published:** 2012-03-19

**Authors:** Demet Demirkol, Dincer Yildizdas, Benan Bayrakci, Bulent Karapinar, Tanil Kendirli, Tolga F Koroglu, Oguz Dursun, Nilgün Erkek, Hakan Gedik, Agop Citak, Selman Kesici, Metin Karabocuoglu, Joseph A Carcillo

**Affiliations:** 1 Department of Pediatric Intensive Care, Bezmialem Vakif University, Faculty of Medicine, Vatan Caddesi, Istanbul, 34093, Turkey; 2 Department of Pediatric Intensive Care, Cukurova University, Faculty of Medicine, Balcali, Adana, 01330, Turkey; 3 Department of Pediatric Intensive Care, Hacattepe University, Faculty of Medicine, Sihhiye, Ankara, 06100, Turkey; 4 Department of Pediatric Intensive Care, Ege University, Faculty of Medicine, Bornova, Izmir, 35100, Turkey; 5 Department of Pediatric Intensive Care, Ankara University, Faculty of Medicine, Sihhiye, Ankara, 06100, Turkey; 6 Department of Pediatric Intensive Care, Dokuz Eylül University, Faculty of Medicine, Inciralti, Izmir, 35340, Turkey; 7 Department of Pediatric Intensive Care, Akdeniz University, Faculty of Medicine, Dumlupinar Bulvari, Antalya, 07059, Turkey; 8 Pediatric Intensive Care Unit, Sami Ulus Education and Training Hospital, Barbur Caddesi, #44, Ankara, 06080, Turkey; 9 Department of Pediatric Intensive Care, Istanbul University, Faculty of Medicine, Millet Cad, Istanbul, 34093, Turkey; 10 Department of Pediatric Intensive Care, Children's Hospital of Pittsburgh, 4401 Penn Avenue, Pittsburgh, PA 15224, USA

## Abstract

**Introduction:**

Hyperferritinemia is associated with increased mortality in pediatric sepsis, multiple organ dysfunction syndrome (MODS), and critical illness. The International Histiocyte Society has recommended that children with hyperferritinemia and secondary hemophagocytic lymphohistiocytosis (HLH) or macrophage activation syndrome (MAS) should be treated with the same immunosuppressant/cytotoxic therapies used to treat primary HLH. We hypothesized that patients with hyperferritinemia associated secondary HLH/sepsis/MODS/MAS can be successfully treated with a less immunosuppressant approach than is recommended for primary HLH.

**Methods:**

We conducted a multi-center cohort study of children in Turkish Pediatric Intensive Care units with hyperferritinemia associated secondary HLH/sepsis/MODS/MAS treated with less immunosuppression (plasma exchange and intravenous immunoglobulin or methyl prednisolone) or with the primary HLH protocol (plasma exchange and dexamethasone or cyclosporine A and/or etoposide). The primary outcome assessed was hospital survival.

**Results:**

Twenty-three children with hyperferritinemia and secondary HLH/sepsis/MODS/MAS were enrolled (median ferritin = 6341 μg/dL, median number of organ failures = 5). Univariate and multivariate analyses demonstrated that use of plasma exchange and methyl prednisolone or intravenous immunoglobulin (*n *= 17, survival 100%) was associated with improved survival compared to plasma exchange and dexamethasone and/or cyclosporine and/or etoposide (*n *= 6, survival 50%) (*P *= 0.002).

**Conclusions:**

Children with hyperferritinemia and secondary HLH/sepsis/MODS/MAS can be successfully treated with plasma exchange, intravenous immunoglobulin, and methylprednisone. Randomized trials are required to evaluate if the HLH-94 protocol is helpful or harmful compared to this less immune suppressive and cytotoxic approach in this specific population.

## Introduction

Hemophagocytic lymphohistiocytosis (HLH) is a life-threatening disorder that can rapidly deteriorate and lead to multiple organ failure (MOF) and death [1,2]. It is classified as primary (familial) or secondary (acquired) [3,4]. Primary HLH is an autosomal recessive disorder caused by a number of different perforin signaling mutations [5]. Secondary HLH is associated with viral, bacterial, fungal, and parasitic infections and malignant disorders [4] in patients without this autosomal recessive disorder. Autoimmune disease-associated HLH is classified as macrophage activation syndrome (MAS) [6].

Hemophagocytic disorders result when critical regulatory pathways responsible for the natural termination of immune/inflammatory responses are disrupted or overwhelmed. Hemophagocytic lymphohistiocytosis is characterized by multisystem inflammation, a reactive process resulting from prolonged and intense activation of antigen-processing cells (macrophages and histiocytes) and CD8^+ ^T cells, and excessive proliferation and ectopic migration of T cells. Studies of cytokine levels in blood and tissue have indicated persistently elevated circulating levels of multiple pro-inflammatory cytokines during symptomatic disease [7-9]. It is currently believed that 'hypercytokinemia' and possibly 'hyperchemokinemia' generated by uncontrolled activation of histiocytes cause MOF.

According to guidelines of the International Histiocyte Society, a diagnosis of HLH requires at least five of the following eight criteria are met: fever, splenomegaly, cytopenias, hypertriglyceridemia or hypofibrinogenemia (or both), hyperferritinemia, elevated soluble interleukin-2 receptor alpha (IL-2Rα), decreased natural killer (NK) cell activity, and hemophagocytosis in bone marrow [4]. Unfortunately, the diagnosis of HLH is complicated by its relatively non-specific clinical presentation. Although hypercytokinemia is a hallmark of HLH, it has also been associated with sepsis, systemic inflammatory response syndrome (SIRS), and multiple organ dysfunction syndrome (MODS) [10,11]. Soluble IL-2Rα is sensitive and specific for HLH [12] but is also elevated in sepsis/MODS/MAS. Of the various laboratory variables available for HLH diagnosis, the most widely used is ferritin. Ferritin is a ubiquitous iron-binding protein that regulates iron storage and homeostasis. The ferritin heavy-chain gene also positively regulates pro-inflammatory cytokine signaling through the nuclear factor-kappa-B pathway [13]. Hyperferritinemia is frequently seen in the intensive care unit, is a marker of a final common pathway of systemic inflammatory response, and is associated with the severity of the underlying disease [14,15]. Bennett and colleagues [15] showed that the hazard ratio of death with peak ferritin of greater than 3,000 ng/mL was 4.32. Hyperferritinemia has also been associated with HLH and many other inflammatory conditions such as sepsis, SIRS, MODS, and MAS [16,17].

According to the International Histiocyte Society guidelines, the treatment for HLH involves an initial 8 weeks of chemoimmune therapy [4]. The immediate aim of chemotherapy in HLH is suppression of the increased inflammatory response and control of cell proliferation. Clinical case series and case reports and animal models suggest that implementation of the HLH protocol has resulted in improved survival in primary HLH; however, the beneficial effect of the protocol for patients with secondary HLH or MAS is questioned. In an effort not to delay treatment in patients with primary HLH, the International Histiocyte Society recommends treating hyperferritinemia-associated secondary HLH/sepsis/MODS/MAS with the same protocol used for primary HLH. In contrast, our hypothesis is that hyperferritinemia-associated secondary HLH/sepsis/MODS/MAS can be successfully treated with a less immunosuppressive and cytotoxic approach than is recommended for primary HLH.

We performed an observational cohort study to evaluate outcome in children with hyperferritinemia and secondary HLH/sepsis/MODS/MAS and compared those who received plasma exchange (PE) with intravenous immunoglobulin (IVIG) or methylprednisone or both with those who received PE with the HLH-94 protocol. We hypothesized that hospital survival would be better with the less immunosuppressive/cytoxic approach.

## Materials and methods

We performed the observational cohort study between December 2005 and April 2011. The study was approved by the ethics committee of the Istanbul Faculty of Medicine (reference number 2011/205-469). Informed consent to participation in the study and to publication of this article was obtained from the guardians of the patients.

Eight centers participated in the Turkish Secondary HLH/MAS Critical Care Study Group. Patients who had secondary HLH and MAS were entered in the study. The patients with primary HLH were excluded from the study. The sample size was estimated *a priori *to be 20 patients with hyperferritinemia and secondary HLH/MAS. A standardized study form, developed by all participating pediatric intensive care centers, was filled in for each patient by the responsible pediatric intensivists from each center. The form consisted of patient age, gender, primary disease, underlying disease, consanguinity, family history of HLH, Pediatric Risk of Mortality III (PRISM III) score, Pediatric Logistic Organ Dysfunction (PELOD) score, numbers of organs involved and the type of organ involvement, microbiological and laboratory data, respiratory support, treatment protocols used, and hospital mortality. The severity of illness was classified by using the PRISM III score and calculated from the most abnormal values in the first 24 hours after admission [18]. Organ dysfunctions were scored according to the PELOD scoring system [19].

The HLH/MAS was diagnosed if five of the following eight criteria were met: (a) fever, (b) splenomegaly, (c) cytopenias (at least two of the following: hemoglobin of less than 9 g/L, platelet count of fewer than 100 × 10^9^/L, and neutrophil count of fewer than 1.0 × 10^9^/L), (d) hypertryglyceridemia (at least 265 mg/dL) or hypofibrinogenemia (not more than 150 mg/L) or both, (e) bone marrow hemophagocytosis, (f) hyperferritinemia (greater than 500 μg/L), (g) increased soluble CD25, and (h) absent NK activity. Patients were not routinely tested for sCD25 levels or NK cell activity; however, all were tested for ferritin levels and bone marrow hemophagocytosis. The patient was classified as having primary HLH if there was a severe clinical presentation without a proven or suspected infection history or metabolic disease and at least one of the following criteria: family history or parental consanguinity, presentation before the age of 2 years, severe clinical presentation with central nervous system involvement, or persistence or recurrence of HLH [4]. Therefore, the children in our study were classified with secondary HLH/MAS because they had suspected or proven infection, no family history or parental consanguinity, were 2 years old or older, had no central nervous system involvement, and had no prior history of HLH. MAS was defined in our cohort in patients who met HLH criteria with an underlying autoimmune disease [5]. SIRS, infection, sepsis, severe sepsis, and septic shock were defined according to the guidelines of the International Pediatric Sepsis Consensus Conference [20]. Treatment failure was defined as progressive disease despite therapy.

The PE treatments were performed by a continuous filtration technique (Prisma; Gambro, Lund, Sweden) in five centers with a polypropylene hollow-fiber plasma filter (TPE 2000; Gambro) and by centrifugation technique (Spectra Optica; CardianBCT, Lakewood, CO, USA, and Com.Tec; Fresenius HemoCare GmbH, Bad Homburg, Germany) in three centers; a Spectra Optia tubing set and a PL-1 kit in a dual-needle set, respectively, were used. Vascular access was obtained with a double-lumen catheter placed percutaneously in a central vein. Total plasma volume (TPV) was calculated manually according to this formula: TPV = total blood volume × (1 - hematocrit). Anticoagulation was achieved with heparin. An infusion of heparin was titrated to achieve an activated partial thromboplastin time (aPTT) of between 50 and 70 seconds. No life-threatening complication related to line placement and PE procedure during the study period was seen. Serum ferritin and lactate dehydrogenase levels and platelet counts were recorded at admission and before and after each PE session.

### Statistical analysis

All statistical analysis were performed by using the Statistical Package for Social Sciences (SPSS 10; SPSS Inc., Chicago, IL, USA). Categorical end points were compared by using the chi-squared test. If continuous variables were normal, they were described as the mean ± standard deviation (*P *> 0.05 in Shapiro-Wilk test), and if the continuous variables were not normal, they were described as the median. The continuous variables were compared by using Mann-Whitney *U *analysis. Factors associated with a *P *value of less than 0.1 in univariate analysis were further evaluated in a multiple regression analysis. A *P *value of less than 0.05 was considered significant in the multiple regression analysis.

## Results

The eight centers identified 34 patients with hyperferritinemia and MODS. Primary HLH was diagnosed in five patients and was treated with the HLH-94 protocol, and the patients had 50% survivals and were excluded from the study. Six patients did not meet five of the eight criteria required for a diagnosis HLH/MAS and were excluded from further analysis. Secondary HLH/MAS was diagnosed in 23 patients, who met criteria for analysis and whose data are presented herein. According to the International Pediatric Sepsis Consensus definitions, 56% (*n *= 13) of these patients also met the diagnostic criteria for septic shock and 9% (*n *= 2) for severe sepsis. The characteristics and clinical presentations of these 23 patients with secondary HLH/sepsis/MODS/MAS are presented in Tables 1 and 2.

**Table 1:** Baseline characteristics of patients with hyperferritinemia and secondary HLH/sepsis/MODS/MAS

Variable	Patients with secondary HLH/MAS (*n *= 23)		
	Mean ± SD	Median	Range
Age, years	7.2 ± 3.6	6.7	2-15
PRISM III-24 score	23.9 ± 12.5	19.5	7-48
PELOD score	26 ± 13.8	24	4-53
Number of dysfunctional organs	4.6 ± 1	5	3-6
	Percentage	Number	
Males/Females	74/26	17/6	
Artificial ventilation	87	20	
Underlying disease	78	18	

HLH, hemophagocytic lymphohistiocytosis; MAS, macrophage activation syndrome; MODS, multiple organ dysfunction syndrome; PELOD, Pediatric Logistic Organ Dysfunction; PRISM, Pediatric Risk of Mortality; SD, standard deviation.

**Table 2:** Diagnostic findings of patients with hyperferritinemia and secondary HLH/sepsis/MODS/MAS

Variable	Secondary HLH/MAS (*n *= 23), percentage (number)
Fever	100 (23)
Splenomegaly	65 (15)
Cytopenia	96 (22)
Anemia	96 (22)
Thrombocytopenia	91 (21)
Neutropenia	44 (10)
Hypertriglyceridemia	87 (20)
Hypofibrinogenemia	39 (9)
Hyperferritinemia	100 (23)
Hemophagocytosis in bone marrow	100 (23)
Survival	87 (20)

All of the patients met five or more of the criteria required for a diagnosis of hemophagocytic lymphohistiocytosis/macrophage activation syndrome (HLH/MAS). HLH, hemophagocytic lymphohistiocytosis; MAS, macrophage activation syndrome; MODS, multiple organ dysfunction syndrome.

**Figure 1:**
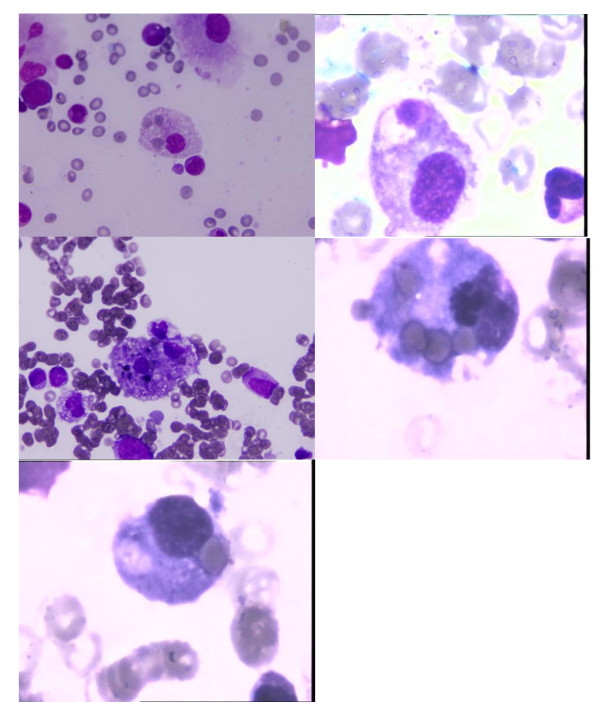
**Bone marrow smears show activated macrophages and hemophagocytosis from bone marrow puncture**. Photomicrographs of a bone marrow aspirate smear show large histiocytes (with vacuolated cytoplasm containing nuclear debris and whole red blood cells). Stain: Wright-Giemsa. Magnification: 1,000×.

Sixty-five percent of the 23 patients (*n *= 15) were classified as having secondary HLH. Thirty-five percent of the patients (*n *= 8) were classified as having MAS: juvenile rheumatoid arthritis (*n *= 6), systemic lupus erytheromatosis (*n *= 1), and polyarteritis nodosa (*n *= 1). All of the patients had hyperferrtinemia as well as hemophagocytosis in their bone marrow aspirate (Figure 1). The diagnostic laboratory variables of the patients with secondary HLH/MAS are presented in Table 3. An underlying disease was present in 78% (*n *= 18) of the patients (Table 1). The precipitating factors in patients with secondary HLH/MAS were viral and non-viral infections in 93% (*n *= 14) of the patients. The microbiological agents were Varicella zoster virus (*n *= 3), *Acinetobacter baumannii*, *Salmonella enteritidis*, *Eschericia coli *(*n *= 2), *Staphylococcus aureus*, *Stenotrophomonas maltophilia*, *Candida albicans*, Epstein-Barr virus, and H1N1 virus infection. In one patient with secondary HLH, no precipitating factors were found. The precipitating factors in patients with MAS were drugs in three patients, namely naproxen sodium, cefepime, and ampicillin-sulbactam. The precipitating factor was infection in one patient with MAS (*Burcholderia cepacia*). In four patients with MAS, no precipitating factors other than autoimmune disease were found.

**Table 3:** Laboratory variables of patients with secondary HLH/sepsis/MODS/MAS

Variable	Mean ± SD	Median	Range
Hemoglobin, g/dL	7.8 ± 2.1	8.1	4.8-15.0
Leukocytes,/mm^3^	10,260 ± 11,402	6,240	400-46,000
Absolute neutrophil count,/mm^3^	7,796 ± 9,148	5,025	0-35.000
Platelet count,/mm^3^	59,221 ± 54,733	51,000	10,000-235,000
PT, seconds	24.8 ± 14.3	21.7	10.3-69.0
aPTT, seconds	42.3 ± 14.5	39	24.7-74.2
Fibrinogen, mg/dL	206 ± 161	171	0-607
Triglycerides, mg/dL	555 ± 348	528	108-1,511
Albumin, g/L	2.6 ± 0.6	2.6	1.4-4.1
Sodium, mEq/L	132.2 ± 7.1	132	120-147
Alanine aminotransferase, U/L	962 ± 2,713	135	21-11,276
Lactate dehydrogenase, U/L	3,721 ± 3,071	2,300	765-100,000
Ferritin, μg/dL	25,313 ± 31,246	6,341	765-100,000

aPTT, activated partial thromboplastin time; HLH, hemophagocytic lymphohistiocytosis; MAS, macrophage activation syndrome; MODS, multiple organ dysfunction syndrome; PT, Prothrombin time; SD, standard deviation.

During the study period, patients with hyperferritinemia and secondary HLH/sepsis/MODS/MAS were assigned to one of four different treatment protocols according to center preference. In protocol 1, the patients (*n *= 8) received PE or intravenous methylprednisolone or both. PE was performed by using a rapid exchange of 1.5 estimated TPVs in the first PE session and then a lower exchange rate with one estimated TPV every 24 hours. Intravenous methylprednisolone was given at 30 mg/kg for 3 days, 20 mg/kg for 2 days, 10 mg/kg for 2 days, 5 mg/kg for 2 days, and then 2 mg/kg. Intravenous methylprednisolone was not used in two patients in protocol 1, because of severe Varicella infection. In protocol 2, the patients (*n *= 9) received PE and IVIG. PE was performed by using 1.5 estimated TPVs every 24 hours, and IVIG was given at a dose of 1 g/kg.

In protocol 3, the patients (*n *= 4) received the HLH-94 protocol (intravenous dexamethasone 10 mg/m^2^, intravenous cyclosporine A 6 mg/kg, or intravenous etoposide 150 mg/m^2^) and IVIG 2 g/kg. If treatment failure was determined, then PE was performed with 1.5 estimated TPVs every 24 hours. In protocol 4, the patients (*n *= 2) received dexamethasone 10 mg/m^2 ^and IVIG 1 g/kg. If treatment failure was determined, then PE was performed with one estimated TPV.

**Figure 2:**
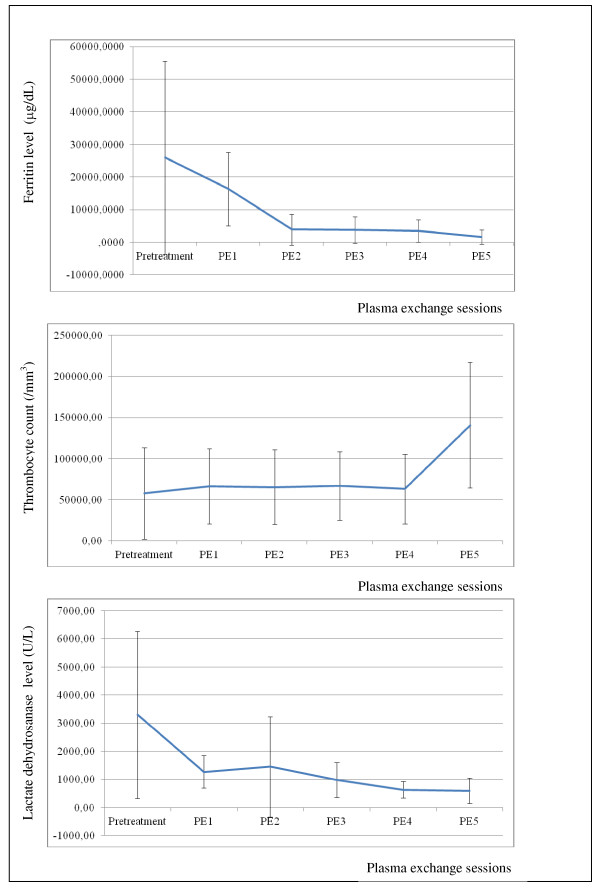
**Change of serum ferritin and lactate dehydrogenase levels and platelet counts after therapeutic plasma exchange (PE) sessions**. The serum ferritin and lactate dehydrogenase levels decreased and platelet counts gradually increased after PE sessions.

In all eight centers, PE was performed in patients with hyperferritinemia and more than two organ dysfunctions. PE was continued until organ dysfunctions resolved, ferritin levels decreased, and platelet count increased greater than 100,000/mm^3^. PE was performed median 5 (1 to 18) times, and PE median doses were 1.5 estimated TPVs and 78 mL/kg (60 to 80). The serum ferritin and lactate dehydrogenase levels decreased and platelet counts increased gradually after each PE sessions (Figure 2).

The overall mortality was 13% (*n *= 3). Mortality rates were 50% (2/4) and 50% (1/2) in the patients with secondary HLH/sepsis/MODS/MAS treated with chemotherapy and dexamethasone-based treatment protocols (protocols 3 and 4, respectively). Despite therapy, two patients died because of progressive MOF and one patient died because of pulmonary bleeding. Two of the non-survivors had a culture-positive infection. There were no deaths in children who were not treated protocols based on dexamethasone, cyclosporin A, or etoposide.

We compared demographic variables, laboratory parameters, and use of treatment protocols as they related to outcome in the survivors and non-survivors (Table 4). There were differences between survivors and non-survivors in regard to gender, serum ferritin levels, and treatment protocols (all *P *< 0.1). In the multiple regression analysis, gender, serum ferritin level, and treatment protocols were independent variables. Survival was best explained by the treatment protocol used (R = 0.55, β = 0.6, *P *= 0.001, 95% confidence interval (CI) 0.1 to 0.33) even after ferritin levels and gender were controlled for. Ferritin levels also predicted survival (R = 0.74, β = 0.4, *P *= 0.004, 95% CI 0.0001 to 0.0008) but gender did not.

**Table 4:** Demographic characteristics and laboratory variables of survivors and non-survivors

	Survivors (*n *= 20)			Non-survivors (*n *= 3)			
	Frequency, percentage		Number	Frequency, percentage		Number	*P*
Females/Males	20/80		4/16	67/33		2/1	0.08
Underlying disease	80		16	67		2	0.6
Mechanical ventilation	85		17	100		3	0.5
	Mean ± SD	Median	Range	Mean ± SD	Median	Range	*P*
Age, years	6.9 ± 3.3	6.7	2-14	8 ± 6.3	6.2	2.8-15.1	0.7
PRISM III-24 score	25.3 ± 13	21	7-48	14 ± 5.1	14	11-21	0.2
PELOD score	27.2 ± 14.5	25	4-53	22.3 ± 9.1	21	14-32	0.7
Number of organs involved	4.5 ± 1	5	3-6	5 ± 1	5	4-6	0.5
Hemoglobin, g/dL	7.5 ± 1.4	8	4.8-8.9	9.6 ± 4.9	8.8	5.2-15	0.4
Leukocytes,/mm^3^	8,814 ± 8,836	6,220	400-26,400	19,900 ± 22,981	11,000	2,700-46,000	0.3
Absolute neutrophil count,/mm^3^	66,688 ± 7,279	4,370	0-23,000	14,816 ± 17,783	8,030	1,450-35,000	0.3
Platelets,/mm^3^	59,255 ± 57,758	48,000	10,000-235,000	59,000 ± 35,510	79,000	18,000-80,000	0.6
PT, seconds	26.4 ± 14.9	24.9	10.3-69	15.3 ± 1.3	15.3	13.9-16.5	0.2
aPTT, seconds	41.9 ± 14.1	39	24.7-74	41.2 ± 17.2	39.4	25-59	0.5
Fibrinogen, mg/dL	201 ± 162	180	0-607	236 ± 180	156	110-443	0.2
Triglycerides, mg/dL	554 ± 349	436	122-1551	559.5 ± 417	638	108-931	0.7
Albumin, g/dL	2.6 ± 0.6	2.7	1.4-4.1	2.8 ± 0.5	2.6	2.4-3.3	0.8
Sodium, mEq/L	131 ± 6	132	120-141	141 ± 8.5	141	135-147	0.3
ALT, U/L	324 ± 585	128	21-2,362	5,751 ± 7,812	1250	227-11,276	0.14
LDH, U/L	3,884 ± 3146	2300	410-11,270	2,327 ± 2,665	2,327	443-4,212	0.6
Ferritin, μg/dL	20,124 ± 27163	5054	765-100,000	40,802 ± 34,583	40,802	16,348-65,256	0.06
Treatment protocols	Frequency	Number		Frequency	Number		0.002
PE and chemotherapy or dexamethasone (protocols 3 and 4)	50%	3/6		50%	3/6		
PE and methylprednisolone or IVIG (protocols 1 and 2)	100%	17/17		None			

ALT, alanine aminotransferase; aPTT, activated partial thromboplastin time; IVIG, intravenous immunoglobulin; LDH, lactate dehydrogenase; PE, plasma exchange; PELOD, Pediatric Logistic Organ Dysfunction; PRISM, Pediatric Risk of Mortality; PT, Prothrombin time; SD, standard deviation.

We further compared demographic variables and laboratory parameters between the 'PE and methylprednisolone or IVIG treated group' and the 'PE and chemotherapy or dexamethasone treated group' (Table 5). There were differences between treatment protocol use in regard to gender, prothrombin time (PT), aPTT levels, and alanine aminotransferase (ALT) levels (*P *< 0.1). In the multiple regression analysis, in which gender, PT, aPTT, ALT levels, and treatment protocol were controlled for, only treatment protocol remained associated with survival (R = 0.84, β = 0.79, *P *= 0.03, 95% CI 0.8 to 1.5).

**Table 5:** Demographic characteristics and laboratory variables of patients with secondary HLH/MAS treated with PE and methylprednisolone or IVIG or with PE and chemotherapy or dexamethasone

	PE and methylprednisolone or IVIG (*n *= 17)			PE and chemotherapy or dexamethasone (*n *= 6)			
	Frequency, percentage	Number		Frequency, percentage	Number		*P*
Males/Females	88/12	15/2		33/67	2/4		0.003
Underlying disease	77	13		83	5		0.6
Mechanical ventilation	89	15		83	5		0.5
	Mean ± SD	Median	Range	Mean ± SD	Median	Range	
Age, years	6.8 ± 3.3	6.5	2-14	7.6 ± 4.8	6.8	2.8-15.1	0.8
PRISM III-24 score	25.6 ± 13.4	25.6	7-48	19.5 ± 8.8	17.5	11-53	0.3
PELOD score	26.2 ± 13.8	24	4-52	27.5 ± 15.	26.5	13-53	0.9
Number of organs involved	4.5 ± 1	5	3-6	4.6 ± 1	5	3-6	0.7
Hemoglobin, g/dL	7.3 ± 1.5	8	4.8-8.9	8.8 ± 3.3	8.3	5.2-15	0.4
Leukocytes,/mm^3^	10,145 ± 9,230	6,270	500-26,400	12,096 ± 17189	6,500	400-46,600	0.9
Absolute neutrophil count,/mm^3^	7,420 ± 7,598	7,420	100-23,300	8,801 ± 13,291	4,475	0-35,000	0.6
Platelet,/mm^3^	51,594 ± 42,767	45,000	10,000-187,000	80,833 ± 81,052	70,000	12,000-235,000	0.6
PT, seconds	29.2 ± 14.68	25	13-69	14 ± 2.1	14.1	10.3-16.5	0.004
aPTT, seconds	45.2 ± 13.1	45.8	30-74	33.2 ± 14	25.7	24.7-59.3	0.05
Fibrinogen, mg/dL	188.3 ± 169	148.5	0-607	206 ± 139	206	110-443	0.2
Triglycerides, mg/dL	515 ± 275	450	122-1,094	654 ± 507	547	108-1,511	0.9
Albumin, mg/dL	2.6 ± 0.7	2.7	1.4-4.1	2.7 ± 0.3	2.6	2.4-3.3	0.9
Sodium, mEq/L	131 ± 6.9	131	120-141	136 ± 6.7	135	130-147	0.1
LDH, U/L	42,10 ± 3306	2,300	750-11,270	2,349 ± 1,934	2,310	410-4,374	0.4
ALT, U/L	310 ± 627	100	21-2,362	3,080 ± 5,466	409	227-11,276	0.03
Ferritin, μg/dL	20,538 ± 28,199	5,109	765-100,000	38,844 ± 38,130	31,718	2,998-97,767	0.2
MV duration, days	18.7 ± 17.4	12	2-65	20 ± 25	5	1-60	0.6
PICU LOS, days	23 ± 18.8	16	4-69	21.6 ± 23	12.9	1-60	0.5
Hospital LOS, days	43.9 ± 33.7	30	11-110	39.2 ± 26.3	41.5	5-70	0.8
	Frequency	Number		Frequency	Number		
Survival	100%	17		50%	3		0.002

ALT, alanine aminotransferase; aPTT, activated partial thromboplastin time; HLH, hemophagocytic lymphohistiocytosis; IVIG, intravenous immunoglobulin; LDH, lactate dehydrogenase; LOS, length of stay; MAS, macrophage activation syndrome; MV, mechanical ventilation; PE, plasma exchange; PELOD, Pediatric Logistic Organ Dysfunction; PICU, pediatric intensive care unit; PRISM, Pediatric Risk of Mortality; PT, Prothrombin time; SD, standard deviation.

## Discussion

The severity of the condition of the patients in our cohort study is illustrated by the median ferritin level of 6,341 μg/dL and the presence of severe MODS/MOF. According to Bennett and colleagues [15], higher levels of ferritin (> 3,000 ng/mL) are associated with increased mortality in a dose response fashion, and Garcia and colleagues [17] reported 100% mortality in children with high ferritin levels and severe sepsis. Additionally, the children in our study had a median of five dysfunctioning organs, for which prognosis is considered very poor [21]. According to Leclerc and colleagues [22], there is a cumulative influence of organ dysfunction and the septic state on mortality of critically ill children. Furthermore, given that over half of our patients had septic shock/severe sepsis, the expected mortality of patients with sepsis in our cohort was 92% [22]. The survival observed in our study was significantly better than expected.

It is of major importance for critical care physicians to be aware that the clinical picture of hemophagocytosis encountered during HLH, sepsis, MODS, and MAS can overlap and share common features. These syndromes cannot be reliably discriminated by using diagnostic criteria devised for HLH [1,16,23,24]. Many patients with HLH will progress to develop MODS, which is also seen in patients with severe sepsis [1,2]. Of the 122 children reported to have HLH in 1996, only 25 met the more strict genetic criteria of familial HLH; the rest of the children were deemed to have secondary HLH. A variety of primary infections were present in 41% of these secondary HLH cases [25]. Hemophagocytosis has been described in 64.5% of 107 autopsies in critically ill medical patients with thrombocytopenia-associated MOF, and all patients with hemophagocytosis had infection [26]. Castillo and Carcillo [16] reviewed the shared clinical similarities between secondary HLH, severe sepsis, SIRS, MODS, and MAS and concluded that these entities could be considered intermediate phenotypes of the same inflammatory process.

According to the International Histiocyte Society guidelines, the treatment of HLH involves chemoimmune therapy. However, some investigators question whether these patients, especially those with sepsis-associated hemophagocytsosis, should receive immunosuppression treatment even when HLH criteria are met. Patients with secondary HLH treated with chemotherapy-based protocols have had only a 55% survival at 3 years, and early mortality was related to hemorrhages and infections [27]. An HLH series of 20 patients showed a mortality rate of 60%, and deaths were attributed to invasive infections in eight cases [28]. In a previous Turkish pediatric cohort, Karapınar and colleagues [2] reported that survival was 43% in critically ill pediatric patients whose HLH and MODS were treated with the HLH-94 2004 protocol. Recently published adult data showed an 89% mortality in patients with virus-associated hemophagocytic syndrome treated with chemoimmune therapy [29]. By contrast, in the present study, 65% of the patients with hyperferritinemia and MODS had active infections and the survival was 100% in the group were not treated with chemotherapeutics or dexamethasone.

All patients in our cohort study received PE therapy. PE is an extracorporeal blood purification technique designed to remove various toxic and inflammatory mediators and to replenish essential compounds via the replacement plasma. PE therapy reverses HLH, possibly by decreasing circulating inflammatory cytokines, including macrophage colony-stimulating factor (M-CSF) [30,31]. There have also been anecdotal reports of patients with secondary HLH/MAS successfully treated with PE [30-32]. In the present study, PE was used as standard care for patients with secondary HLH/MAS to control hypercytokinemia. Several studies favor the use of PE in severely ill septic patients with MODS and thrombocytopenia-associated MOF [33-35]. Stegmayr and colleagues [36] reported a beneficial effect of PE as supportive therapy in patients with progressive disseminated intravascular coagulation and MODS, especially if septic shock was present. In the present study, mortality was detected only in patients who received treatment with cyclosporine, dexamethasone, and/or etoposide. Thus, any beneficial effects of PE might not override the impact of chemotherapeutics in children with secondary HLH/sepsis/MODS/MAS.

The addition of intravenous methylprednisolone and immunoglobulins as anti-inflammatory agents may be less toxic than the addition of dexamethasone, cyclosporine A, and/or etoposide. Intravenous dexamethasone is cytotoxic for lymphocytes and inhibits expression of cytokines and differentiation of dendritic cells. Since dexamethasone crosses the blood-brain barrier better than methylprednisolone, it offers the advantage of suppressing central nervous system inflammation for patients with primary HLH; however, the immunosuppressive potency of dexamethasone may be deleterious in patients with sepsis. Hurwitz and colleagues [37] reported the occurrence of fatal or near-fatal sepsis in 16 of 38 children with newly diagnosed acute lymphoblastic leukemia (ALL) treated with a new induction regimen that differed from its predecessor in that it administered long-term high-dose dexamethasone. The investigators concluded that the substitution of dexamethasone for prednisone or methylprednisolone in an otherwise intensive conventional induction regimen for previously untreated children with ALL resulted in an alarmingly high incidence of septic episodes and deaths [36]. In contrast, high-dose intravenous methylprednisolone treatment did not increase mortality. Etoposide and cyclosporin A also have potent immunosuppressive and cytotoxic effects compared with IVIG. Immunoglobulins probably act, in part, by providing cytokine- and pathogen-specific antibodies. Hence, in children with active infection, it may be better not to use agents that impair the host's ability to fight off infection.

There are several limitations to consider when evaluating our study. By nature of the cohort design, this is not a randomized controlled study. A randomized trial comparing the two treatment strategies are needed. Second, since all patients received PE therapy, the observations in this study may or may not apply to children who do not receive PE therapy as part of the approach to secondary HLH/sepsis/MODS/MAS. Third, we did not routinely measure NK cell activity or sCD25, nor did we address perforin signaling genetics. Therefore, our findings should not be applied to children with primary HLH and homozygous perforin signaling mutational defects. Our findings should be considered only in children who either are heterozygous or lack these mutations. As Behrens and colleagues [38] recently reported, an experimental model of repeated Toll-like receptor-9 (TLR-9) stimulation with interferon-gamma led to HLH/MAS after endotoxin exposure in mice without any perforin signaling mutations. Our children may be better represented by this experimental model of acquired HLH/MAS disease than by the well-established congenital perforin signaling mutation knockout mouse models of primary HLH [39]. One should strictly use our definitions of secondary HLH/MAS (having suspected or proven infection and no family history or parental consanguinity, being 2 years old or older, having no central nervous system involvement, and having no prior history of HLH) when considering the applicability of our findings to one's practice. Finally, there are no data between outcome and gender association in patients with secondary HLH/MAS. The female preponderance seen in mortality in the present study needs further investigation.

## Conclusions

This cohort study supports the beneficial effect of PE and less immunosuppressive and cytotoxic methylprednisone and IVIG therapies in patients with hyperferritinemia and secondary HLH/sepsis/MODS/MAS. No significant difference was seen in outcome between the more and less immunosuppressive groups, however the less immunosuppressive therapy may have less toxic effects than observed with the HLH-94 protocol and should be considered. Until a randomized trial is performed, one could consider this approach for the treatment of secondary HLH/MAS with MODS, especially in patients with sepsis-associated HLH/MAS.

## Key messages

• Hyperferritinemia-associated secondary hemophagocytic lymphohistiocytosis (HLH)/sepsis/multiple organ dysfunction syndrome (MODS)/macrophage activation syndrome (MAS) can be successfully treated with a less immunosuppressive approach than is recommended for primary HLH.

• Plasma exchange may be an important therapeutic tool in the pediatric field for treatment of secondary HLH/MAS with MODS.

## Abbreviations

ALL: acute lymphoblastic leukemia; ALT: alanine aminotransferase; aPTT: activated partial thromboplastin time; CI: confidence interval; HLH: hemophagocytic lymphohistiocytosis; IL-2Rα: interleukin-2 receptor alpha; IVIG: intravenous immunoglobulin; MAS: macrophage activation syndrome; MODS: multiple organ dysfunction syndrome; MOF: multiple organ failure; NK: natural killer; PE: plasma exchange; PELOD: Pediatric Logistic Organ Dysfunction; PRISM: Pediatric Risk of Mortality; PT: prothrombin time; SIRS: systemic inflammatory response syndrome; TPV: total plasma volume.

## Competing interests

The authors declare that they have no competing interests.

## Authors' contributions

DD is the guarantor of integrity of the entire study and participated in study concepts and design, definition of intellectual content, data collection, literature research, data analysis, and manuscript preparation, editing, and review. DY, BB, BK, TK, TFK, OD, NE, HG, AC, SK, and MK participated in study design, definition of intellectual content, data collection, and manuscript preparation, editing, and review. JAC participated in study concepts and design, definition of intellectual content, data collection, literature research, data analysis, and manuscript preparation, editing, and review. The authors read and approved the final manuscript.
